# New Cutoff Point for Platelet Count/Spleen Diameter Ratio to Predict Esophageal Varices in Patients with Hepatitis C Virus-related Hepatic Cirrhosis

**DOI:** 10.5005/jp-journals-10018-1154

**Published:** 2016-07-09

**Authors:** Walid El-Sherbiny, Mohamed Elegezy, Shaker Wagih Shaltout

**Affiliations:** 1 Department of Tropical Medicine, Mansoura University, Mansoura, Egypt

**Keywords:** Cirrhosis, Platelet count, Spleen diameter, Varices.

## Abstract

The esophagogastroduodenoscopy (EGD) with all its hazards remains the gold standard screening tool for esophageal varices. Noninvasive tools have been proposed and studied to replace the EGD. Platelet count (PC)/spleen diameter (SD) ratio as a noninvasive tool for predicting the presence of esophageal varices was proposed and studied in many previous studies.

**How to cite this article:**

El-Sherbiny W, Elegezy M, Shaltout SW. New Cutoff Point for Platelet Count/ Spleen Diameter Ratio to Predict Esophageal Varices in Patients with Hepatitis C Virus-related Hepatic Cirrhosis. Euroasian J Hepato-Gastroenterol 2015;5(2):136-137.

Dear Editor,

The esophagogastroduodenoscopy (EGD) with all its hazards remains the gold standard screening tool for esophageal varices. Noninvasive tools have been proposed and studied to replace the EGD. Platelet count (PC)/spleen diameter (SD) ratio as a noninvasive tool for predicting the presence of esophageal varices was proposed and studied in many previous studies. In this communication, we enrolled a total of 62 Egyptian cirrhotic patients at the Tropical Medicine Department of Mansoura University Hospital, Egypt. All of these patients underwent screening endoscopy for esophageal varices. Demographic, clinical, biochemical and ultrasonographic parameters were recorded. Platelet count (mm^3^) and spleen diameter (mm) was assessed in all patients. The etiology of cirrhosis in all included patients was hepatitis C virus (HCV). The data were assessed and receiver operating characteristic curves were also applied. The accuracy of the PC/SD in predicting the presence of esophageal varices was evaluated. After analysis of data we found that the PC/SD ratio has a sensitivity of 72.7% and a specificity of 67% at cutoff ≤638 for detection of esophageal varices with a positive predictive value of 84.2%, and a negative predictive value of 50%. A significant correlation between the PC/SD ratio and degree of varices (Spearman’s rho—0.582; p ≤0.001) was also reported.

The development of portal hypertension (PH) is a crucial event in the evolution of cirrhosis and is defined by an increase in the portal pressure above the normal range of 1 to 5 mm Hg, as measured by the hepatic vein pressure gradient (HVPG). When the HVPG increases further to ≥10 mm Hg, PH is defined as clinically significant.^1^ The occurrence of clinically significant portal hypertension (CSPH) is a crucial turning point in the natural history of cirrhosis, because it opens the way to the development of esophageal varices and hepatic decompensation.^2^ Bleeding from esophageal varices is a life-threatening condition with an incidence of 5 to 15% in patients with liver cirrhosis and mortality rates of at least 20%.^3^ Although EGD is the gold standard tool for diagnosing esophageal varices, the EGD is an invasive tool with many hazards and there is a great interest in identifying a noninvasive tool to replace it. Platelet count/ spleen diameter ratio as a noninvasive tool for predicting the presence of varices was proposed by Giannini et al.^4^ The initial results by Giannini et al were supported by many other studies.^4-7^

In this article, we reported a new cutoff point for PC/SD ratio to predict esophageal varices in patients with HCV related hepatic cirrhosis that is endowed with moderately acceptable sensitivity and specificity.
